# Mobile Phones As Surveillance Tools: Implementing and Evaluating a Large-Scale Intersectoral Surveillance System for Rabies in Tanzania

**DOI:** 10.1371/journal.pmed.1002002

**Published:** 2016-04-12

**Authors:** Zacharia Mtema, Joel Changalucha, Sarah Cleaveland, Martin Elias, Heather M. Ferguson, Jo E. B. Halliday, Daniel T. Haydon, Gurdeep Jaswant, Rudovick Kazwala, Gerry F. Killeen, Tiziana Lembo, Kennedy Lushasi, Alpha D. Malishee, Rebecca Mancy, Matthew Maziku, Eberhard M. Mbunda, Geofrey J. M. Mchau, Roderick Murray-Smith, Kristyna Rysava, Khadija Said, Maganga Sambo, Elizabeth Shayo, Lwitiko Sikana, Sunny E Townsend, Honorathy Urassa, Katie Hampson

**Affiliations:** 1 Ifakara Health Institute, Ifakara, Morogoro, Tanzania; 2 Boyd Orr Centre for Population and Ecosystem Health, Institute of Biodiversity, Animal Health and Comparative Medicine, College of Medical, Veterinary and Life Sciences, University of Glasgow, Glasgow, United Kingdom; 3 School of Computing Science, University of Glasgow, Glasgow, United Kingdom; 4 Ministry of Health and Social Welfare, Dar es Salaam, Tanzania; 5 Sokoine University of Agriculture, Department of Preventative Veterinary Medicine, Morogoro, Tanzania; 6 Liverpool School of Tropical Medicine, Department of Vector Biology, Liverpool, United Kingdom; 7 Ministry of Agriculture, Livestock and Fisheries, Dar es Salaam, Tanzania; 8 World Health Organization, Country Office, Dar es Salaam, Tanzania

## Abstract

Katie Hampson and colleagues describe their experience of developing and deploying a large-scale rabies surveillance system based on mobile phones in southern Tanzania.

**Summary Points**

- Surveillance is critical to manage preventative health services and control infectious diseases. Integrated surveillance involving public health, veterinary, and environmental sectors is urgently needed to effectively manage zoonoses and vector-borne diseases. However, most surveillance in low-income countries is paper-based, provides negligible timely feedback, is poorly incentivised, and results in delays, limited reporting, inaccurate data, and costly processing.
- The potential of mobile technologies for improving health system surveillance has been demonstrated through small-scale pilots, but large-scale evaluations under programmatic implementation remain rare.
- An intersectoral mobile-phone–based system was developed and implemented for rabies surveillance across southern Tanzania. Since 2011, the system has facilitated near real-time reporting of animal bites and human and animal vaccine use (almost 30,000 reports) by over 300 frontline health and veterinary workers across a catchment area of 150,000 km^2^ with >10 million inhabitants, improving data quality, timeliness, and completeness while reducing costs.
- The surveillance system infrastructure is a platform that can be further developed to improve services and deliver health interventions; for example, generating automated personalized text messages (SMS) to alert patients to their vaccination schedules improved their compliance with regimens. Other interventions targeting patients and health workers can now be implemented easily.
- The system has become an integrated, popular, and valuable tool across sectors, used routinely throughout southern Tanzania to evaluate the impacts of rabies control and prevention activities and to improve their management, directly informed by the experiences of frontline users.
- We discuss challenges encountered during development and deployment, how we overcame these, and our recommendations for scaling up mobile-phone–based health (mHealth) interventions in low-income countries.

## Introduction

There is huge potential for mobile technologies to improve health care and public health service delivery, especially in resource-poor settings [1–3]. Mobile technologies are ideally suited to surveillance, a fundamental component of health systems critical for measuring the progress of disease control and prevention measures, for appropriate targeting of resources, and for elimination of infectious diseases [4]. Successful surveillance depends on timely and comprehensive gathering of information to assess disease status, determine appropriate control strategies, and monitor their impact. In low- and middle-income countries (LMICs) where infrastructure and communication channels are limited, surveillance poses substantial challenges [5].

Mobile phones are cheap and ubiquitous, with massive growth globally, especially in sub-Saharan Africa [6]. Mobile-phone–based health applications are proliferating rapidly [1] and there are persuasive reasons why mobile technologies offer such potential [7]. They can be used at low cost to deliver scalable interventions, to tailor and personalize care [2], and importantly, to support direct communication between frontline workers, programme managers, patients, and communities. In LMICs, phones provide a means of overcoming structural barriers to access [3] and can empower workers in remote, isolated communities where infrastructure and resources are lacking. However, few mobile-phone–based health systems have been implemented across large spatial scales or evaluated in terms of their usability and impact in LMICs, where there is arguably the most to be gained. We describe the implementation and evaluation of a large-scale mobile-phone–based system used in the context of rabies surveillance in southern Tanzania.

Rabies is a fatal disease that kills thousands of people every year in LMICs, where it is primarily spread by domestic dogs [8]. Following a bite, human rabies deaths can be prevented through prompt administration of post-exposure prophylaxis (PEP), which involves a course of vaccinations administered over several weeks, together with immunoglobulin administration for high-risk exposures [9]. More proactively, the risk of exposure can be reduced and the disease ultimately eliminated through well-implemented mass vaccination programmes for dogs [10]. Surveillance typically requires intersectoral collaboration, especially for zoonoses. For example, in the context of rabies, health workers need to report animal bites to veterinary officers to trigger outbreak investigations, and veterinarians need to alert medical authorities to exposure risks from animal rabies cases. Defining institutional responsibilities for collecting and compiling surveillance information and maintaining effective communication across sectors and hierarchies pose challenges. Solutions that address rabies surveillance needs should therefore have wide applicability across a variety of health applications and other societal needs.

**Fig 1:**
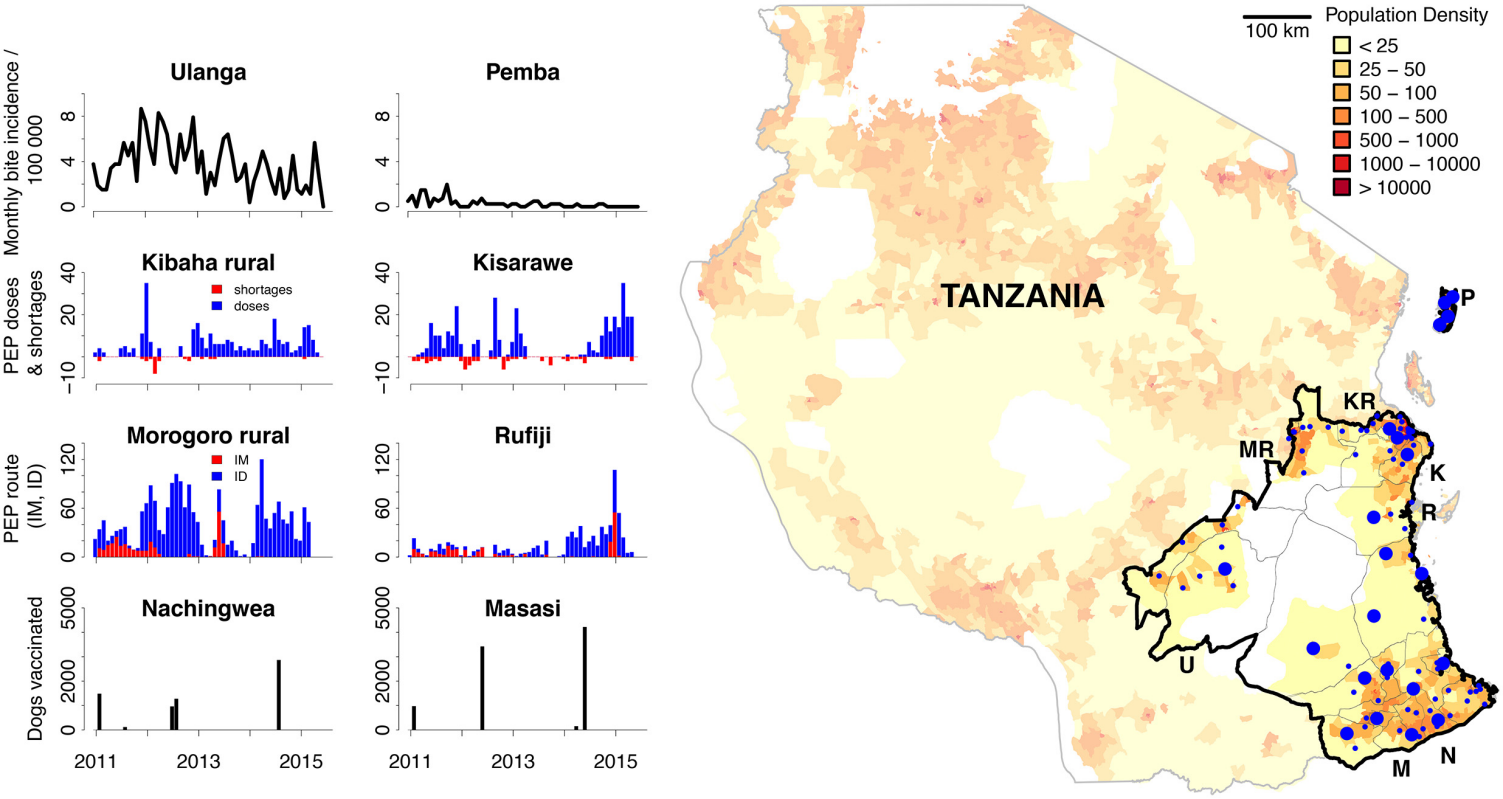
The mobile-phone–based surveillance system. In the map, blue dots represent facilities that provide post-exposure prophylaxis (PEP) and report using the surveillance system (large dots represent hospitals, small dots represent health centres). The map is shaded by population density with wildlife-protected areas in white. The panels illustrate example surveillance data (S1 Data) from different districts that are annotated on the map by their initials. These data show monthly incidence of bite patients per 100,000 people on Pemba Island (P) and Ulanga (U), PEP use and shortages for Kibaha rural (KR) and Kisarawe (K), progress switching from intramuscular (IM) to intradermal (ID) administration of PEP for Morogoro rural (MR) and Rufiji (R), and numbers of dogs vaccinated each month for Nachingwea (N) and Masasi (M).

Our mobile-phone–based surveillance system was designed for frontline health workers to report patients seeking PEP or presenting with clinical signs of rabies and for livestock field officers to report mass dog vaccination campaigns and suspected animal cases. Operating routinely since 2011, the system is currently used by over 300 health workers and livestock field officers across 120 health facilities and 26 veterinary offices (Fig 1) and supports a WHO-coordinated, government-led rabies control programme across southern Tanzania. We discuss challenges of the design, development, implementation, and evaluation of this system; how they were overcome; and lessons learned for scaling up mobile-phone–based health (mHealth) systems in LMICs.

## Development and Deployment of the Surveillance System

This project was conceived during an outbreak of rabies in the Kilombero Valley, southern Tanzania, in 2007. Researchers at Ifakara Health Institute (IHI) were alerted to human rabies deaths and local panic and acute distress amongst families unable to obtain PEP. One medical officer commented: “We have people coming who have been bitten… We keep asking for vaccine but it takes a long time to come and when it comes we only receive ten vials, which is used up in a day… We have to send them away; sometimes they go to Dar es Salaam, but sometimes we don’t know what they do.”

Subsequent research estimated the burden of rabies in these communities, revealing recurring shortages of PEP, as well as considerable economic costs and barriers to those seeking PEP [11]. Meetings with district, regional, and national medical and veterinary personnel revealed frustrations at all levels concerning provision of these life-saving vaccinations.

We discussed the design and development of a prototype mobile-phone–based system with stakeholders to address the highlighted problems, drawing on experience of previous pilot mHealth projects and developers in East Africa [12,13]. In 2010, the WHO and government of Tanzania secured funding for a large-scale rabies control programme across southern Tanzania, as part of a multi-country initiative [14]. Existing paper-based surveillance in the health sector was insufficient for timely evaluation of this control programme, whilst surveillance for monitoring rabies control in the veterinary sector was absent. We therefore expanded the prototype from the Kilombero Valley to the 28 districts of the control programme, covering a catchment area of 150,000 km^2^ and serving >10 million inhabitants (Fig 1).

**Fig 2:**
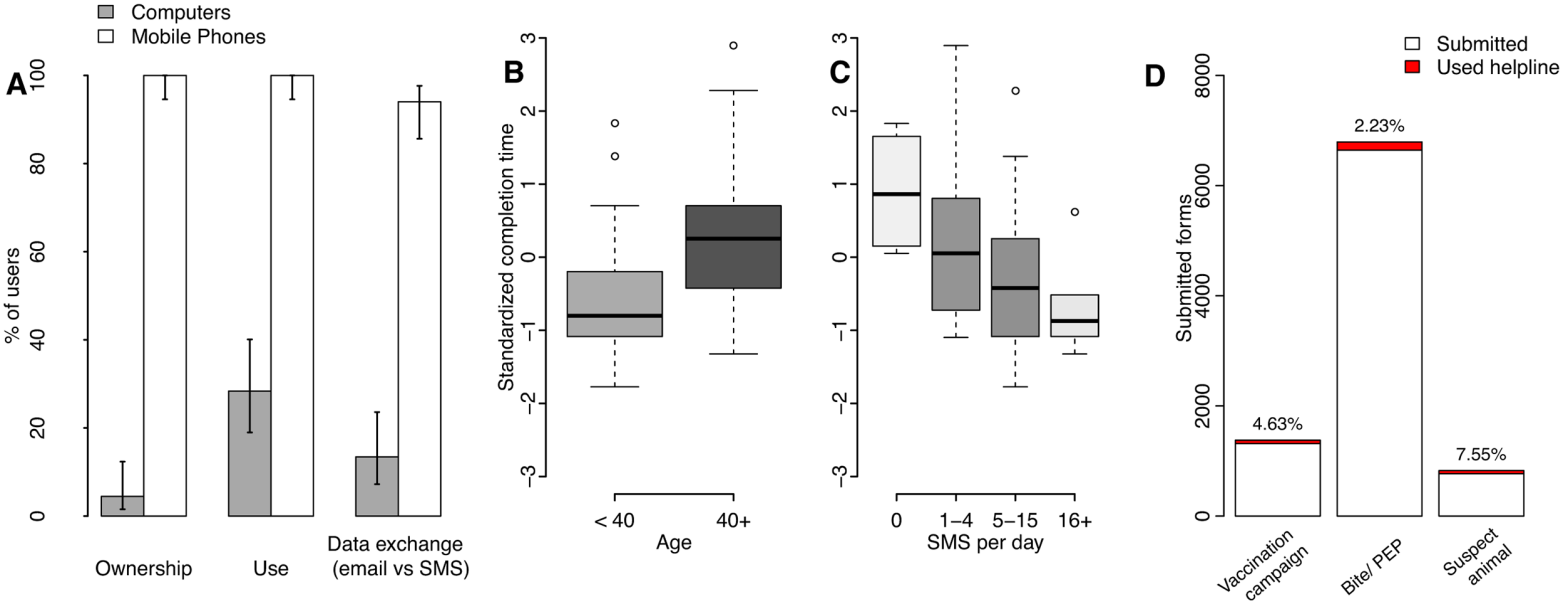
Mobile phones as potential tools for surveillance in Tanzania. (A) Access and use of mobile phones versus computers by surveillance system users and 95% confidence intervals. The effects are shown of user (B) age and (C) self-reported use of text messaging (short message service or SMS), on the standardized time to complete surveillance forms on mobile phones, with boxes shaded in proportion to the sample size in the group (S2 Data). Time to completion in minutes was standardized by computing z-scores by sector, because forms used by health workers for recording bite patients were longer than forms used by livestock field officers to record mass dog vaccination campaigns (S3 Table, S1 Text). (D) Number and percentage of mobile phone form submissions where helpline support was used (<8% overall and <3% for the most commonly used form, that for bite patient records, data in S1 Table). Additional forms submitted by staff involved in system development and therefore familiar with the mobile phone application were excluded.

**Fig 3:**
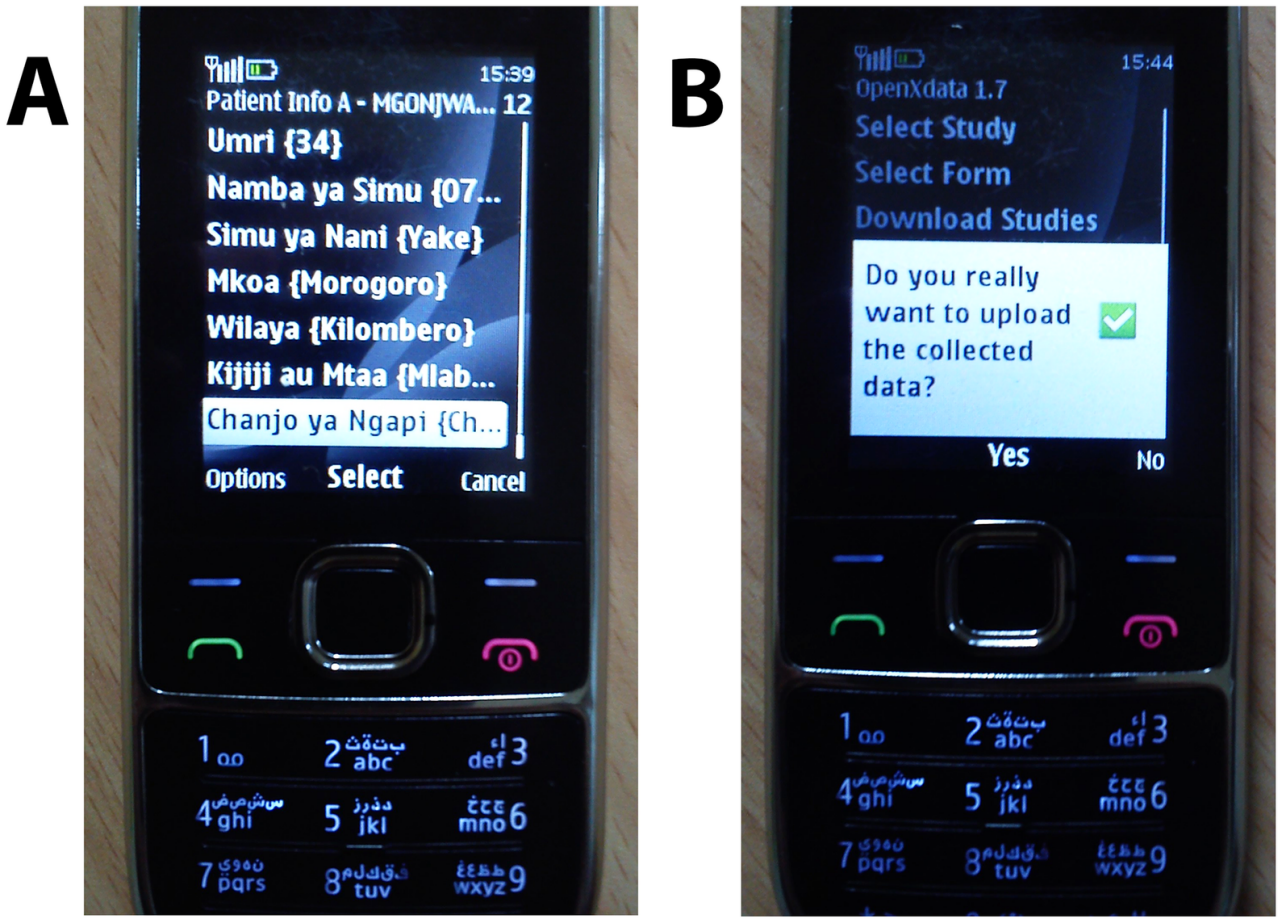
Example of mobile phone surveillance application. Mobile phone interface showing form being (A) completed for an example bite patient and (B) submitted.

Most health and veterinary facilities in southern Tanzania had no Internet access and unreliable power, but mobile phone network coverage was widespread and local staff already owned phones (Fig 2A, S2 Data). The system was developed to use the Global System for Mobile communication (GSM) that enables an Internet connection to mobile phones through the General Packet Radio Service (GPRS), even in rural settings where 3G connections are not available. The system employs a data-entry application (openXdata) on Java-enabled mobile phones (Fig 3) and uses an http protocol to send data to a server running a MySQL database for storage and management. The application only needs to be connected to the remote server for data transfer; user authentication as well as data entry and validation occurs on the mobile phone, where data can be saved until submission.

Forms for phone-based data entry largely adopted formats of existing paper registers used in health facilities for reporting bites, PEP use and rabies deaths. New forms were designed with livestock officers to report suspect animal cases, diagnostic samples submitted to laboratories, and operational details of vaccination campaigns. Forms were designed to minimize free typing, for example, by using radio buttons for multi-choice selection or drop-down menus (Fig 3). The application interface was adapted for local use, with information in the *Kiswahili* language, and prompts running across the phone screen to assist users [15]. Forms included an option for users to send feedback. Users from the two sectors were trained together to improve familiarity amongst veterinary and medical officers of their respective roles and responsibilities, then registered and provided with configured phones (Fig 3). Prior to deployment, pilot data were collected to check system functioning and were reviewed for quality assurance.

## Surveillance In Action

Since establishing the system in 2010, we have registered over 450 users from the human and animal health sectors stationed at the four health facilities per district that provide PEP through the control programme and all the district livestock offices. However, accounting for staff turnover and relocation, we maintain a user base of around 300 active users. In the five years since 2011, 29,595 reports were submitted. Most of these reports recorded bite patients seeking PEP (14,565 records, 49%), detailing visits of approximately 5,800 patients, who on average received 2.3 vaccine doses. Village-level vaccination campaigns accounted for 11,142 records (37%).

By generating disaggregated and spatially localized data, the surveillance system allows detailed monitoring and evaluation of this large-scale control programme across sectors (Fig 1, S1 Data). Overall, the data show progressive increases in coverage and extent of mass dog vaccinations and also identify gaps in coverage. Incidence of bite patients seeking PEP declined substantially (>50%, Fig 1), and rabies has been eliminated from Pemba Island [16]. These shared successes reported through stakeholder meetings have helped to reinforce the benefits of working across sectors and also highlight where action is needed. For example, the data reveal substantial improvements in health service provision overall, whilst allowing quantification of previously overlooked PEP supply shortages in some locations (Fig 1).

Feedback sent by users from their phones provided a powerful mechanism for identifying and communicating previously overlooked problems. For example, two health workers reported, “I advise you to send vaccines to Mvomero district [outside the study area], because patients are travelling from there to our clinic,” and “We remain with only one vial for tomorrow, and will have to turn away bite victims if we don’t receive more.”

Human rabies cases (42 reported) reflected issues with PEP supply, and limited awareness of the need for PEP, as illustrated by the following report: “He was bitten by a dog two months ago but only now comes for treatment, but it is too late as he already is showing symptoms.”

Such feedback submitted directly from frontline workers prompted changes to training and the distribution and supply of PEP and phones to underserved areas without PEP access. More generally, users reported being more aware of rabies and the need to administer PEP to bite victims, suggesting improved service provision. They were also able and frequently did redirect patients to other centres providing PEP when shortages occurred.

Comments mostly indicated that livestock officers were satisfied with vaccination campaigns (e.g., “the work went very well,” “good,” “excellent”) but revealed difficulties in some areas: “The activity in this community was difficult because residents are afraid of vaccinations.”

A valuable feature of the system was the ability to send automated SMS (short message service, commonly known as a “text” message) reminders to patients due for further PEP doses. With four visits to health facilities over a one-month period required for the full PEP course, compliance of patients is often low, which can have deadly consequences because prevention of rabies is not ensured. Compliance with PEP regimens was significantly higher for patients following the implementation of automated reminders in comparison to patients attending clinics prior to this (odds ratio [OR] = 1.58, 95% confidence interval [CI] = [1.20–1.95], *p* < 0.001, S2 Table), and this effect was consistent, irrespective of age, phone access (own/family or friend/neighbour), or location (urban/rural).

In summary, the system allowed many inadequacies of surveillance systems in LMICs to be addressed [17,18] by providing accurate and timely information to improve health service provision and disease control activities. For rabies, ensuring PEP availability still requires work (7% of patients failed to obtain PEP) but the system infrastructure and timely data submission provides a platform for developing and evaluating future improvements (Box 1). Furthermore, the system can be adapted far more generally. For example, it has already been modified to monitor maternal health interventions, as well as malaria vector populations elsewhere in Tanzania.

**Box 1. Recommendations for mHealth Scale-Up**

- The user experience is critical and usability should be prioritized over technological advances—many LMIC users are familiar only with simple handsets (>99% of our users). Given the pace of technological advances, interoperable platforms compatible with a range of handsets are imperative [22].
- Cross platform applications that users can download to their own phone can reduce direct costs and phone losses (we witnessed high staff turnover/relocation and phone loss), increase sustainability, and incentivise use [25]. We were often requested to install the applications to users’ personal phones and found this to be a very satisfactory solution.
- Sufficient resources need to be allocated to user training, support, and feedback, which are vital to the user experience. Support personnel were able to rapidly trace and solve difficulties and help maintain standards, and their regular communication helped motivate users, while recognition of their submitted data and feedback were incentives in themselves.
- Competitive contracts with mobile phone service providers for large user groups should reduce costs. These should also facilitate the use of helpline services and cross-sectoral communication by making data upload and calls free between users; this was frequently requested from users. Public–private partnerships could be further explored to reduce costs.
- Systems should be developed by local personnel with an operational understanding of local health systems and cultural sensitivities because they are invested in the system’s success and are essential to its sustainability, flexibility, and future development [17,26].

**Recommendations for improvement of our mobile-phone–based surveillance identified from stakeholder meetings and user feedback**

- SMS alerts of vaccine shortages, human deaths, and rabies outbreaks; reminders to users of scheduled tasks and feedback on submissions to encourage reporting.
- Automated and dynamic reporting to local, regional, and national stakeholders to enhance update and use of information for disease control and prevention, including targeted programme management, e.g., prompts to repeat vaccination campaigns if coverage targets not achieved.
- Direct installation of the application to users’ own phones to reduce phone losses.
- Adaption of the application to smartphones in addition to Java-enabled phones.
- Migration to cloud storage to overcome server maintenance and power issues.
- Integration into information systems (DHIS2) being adopted within the Tanzanian health sector [27].
- Free rabies phone helpline for public and closed user group contract for free communication between surveillance users, and to support intersectoral and district and regional coordination of control and prevention activities.

## System Usability and Evaluation

We used observations of 40 health workers and 27 livestock officers to examine factors affecting system usability and support required. These users all had mobile phones and almost 95% reported daily SMS use. Only 4% owned computers and 14% had an email address (Fig 2A, S2 Data). After only ten minutes of training, most users could log on without problems. We considered the observed time to fill in and submit a form as an indicator of usability that captures data entry abilities and need of assistance. On first use, users took approximately ten minutes to complete their respective form. General familiarity with phones, SMS and the Internet all facilitated initial use, explaining 47% of the variation in time to complete forms, with SMS use explaining most variation (full details in S1 Text, S3 Table, S2 Data). Age was a useful alternative predictor of potential difficulties for first-time users (Fig 2B). SMS use (Fig 2C) and phone ownership duration accounted for ~25% of the variation, suggesting that increasing familiarity should partially compensate for age-related difficulties. Less than 1% of users owned smartphones (as of January 2015). The important role of interface familiarity therefore suggests that employment of more advanced technologies may currently have disadvantages in terms of usability.

In practice, most users were able to use the system effectively and did not encounter any infrastructure issues, but a few (<2%) had network problems from unreliable power supply to mobile towers and reported issues with charging phones (<3%). In case of problems with phones, replacements were issued. A user helpline staffed by surveillance personnel helped users overcome challenges, and helpline logs recorded difficulties encountered. Once users were trained and submitting data regularly, from January 2011 onwards, the helpline was accessed for only 3% of all submissions (2.2%–7.6% depending upon form type, with greater difficulties encountered for less frequently used forms, Fig 2D, S1 Table). Occasionally, users were identified as reporting infrequently and were reminded of submission steps, or new users required training due to staff turnover or relocation, but over-the-phone support was usually sufficient.

We evaluated case detection capacity of human rabies cases and exposures, timeliness of reporting, and completeness of surveillance according to standard methods [19]. Monthly reports of bite patients submitted by phone (2011–2013) were over 400% higher than reports compiled from paper records (2005–2010) (S4 Table). These differences likely reflect increased recording of bites and PEP administration—which if not available may have led to health workers neglecting to record bites—and increased reporting from local to central levels, as opposed to increased rabies incidence. Paper records were entirely lacking from some regions despite health workers recalling people dying of rabies and bite victims attending clinics. Physical collation of paper records was often delayed for many months or never occurred, and was stated as a major obstacle for restocking of PEP. In contrast, most mobile phone data were submitted promptly, typically within one week, with delays mostly due to heavy workloads and/or limited time, but also occasionally the result of unreliable power, phone loss (6% lost per annum), or user relocation (6% turnover per annum).

Data reported by phone were validated on entry, with error message prompts to ensure quality, and were more complete than paper records (97%–100% versus 70%–96% for paper), which frequently lack age, gender, locations, and dates and require several manual processing steps that are prone to human error and data loss [17,19,20]. Paper records are aggregated to district level on coarse (annual) timescales, whereas the mobile phone system provides near real-time data at much finer, village-scale spatial resolution, with potential to dramatically improve outbreak detection and response. Moreover, the implementation of the system generated interest in rabies and strengthened relationships between local health and veterinary workers who had trained together, catalysing contact and even resulting in the carrying out of joint investigations facilitated by phone support from surveillance staff. These investigations identified suspect rabies victims who did not attend medical facilities and would ordinarily not have been recorded as rabies deaths. However, some users complained about lack of resources for outbreak investigations, sample collection, and shipment, which is an ongoing limitation for rabies surveillance.

Infrastructure and personnel costs for paper-based surveillance were lower than for the mobile phone-based system, but paper-based surveillance required costly processing and transportation (S5 Table). Overall, annual costs for establishing and implementing the mobile phone system were 15% lower than paper-based costs (~US$3,700 versus US$4,300 per district serving an average of 300,000 people, of which running costs constituted ~US$1,430 versus US$3,320), demonstrating that mobile-phone–based surveillance is affordable even in LMICs. However, care should be taken in extrapolating to other contexts because scale and terrain affect training and distribution costs. Another caveat is that, in practice, paper-based surveillance costs may appear lower simply because many records were never submitted and collated.

We sought more general feedback through, for example, regular attendance at government meetings to understand stakeholder requirements, and we adapted the system wherever possible, e.g., adjusting phone settings to retain data for reference. User feedback sent via phones revealed potential areas for improvement of rabies prevention and control activities as well as service delivery system shortcomings, whilst stakeholder discussions during a workshop in October 2013 led to suggestions for future system development (Box 1).

## Conclusion

Through the development, implementation, and evaluation of this mobile-phone–based surveillance system in southern Tanzania, we have demonstrated the considerable value and feasibility for mobile technologies to improve health systems, services, and outcomes in LMICs. Frontline health and veterinary workers needed only minimum education and experience to operate the system, with usability mostly affected by their previous level of experience with mobile phones. The system has facilitated ongoing data collection across large programmatic scales, greatly improving data quality, timeliness, completeness, and cost-effectiveness. The resulting surveillance is being used to evaluate the impacts of ongoing rabies control activities and improve their management, directly informed by the experiences of frontline users. As a result, the system has become an integrated, popular, and valuable tool within the health and veterinary sectors in southern Tanzania.

MHealth has been criticised for the proliferation of pilot studies with little coordination and programmatic evidence of effectiveness to inform scale-up [3,21,22]. Although the pace of ongoing technical advances is exciting, with mHealth piggybacking on this momentum, the goal for mHealth now is to move beyond pilots to sustainable integration within health systems and culture [20,23,24]. Box 1 lists technical and acceptability challenges of large-scale mHealth programmes, possible solutions, and further opportunities for development as drawn from experiences of our system. For neglected zoonotic diseases, such as anthrax, cystic echinococcus, leishmaniasis, human African trypanosomiasis, Rift Valley fever, and plague, that cause considerable mortality and morbidity in low-income countries, there is a real unmet need for integrated intersectoral surveillance systems, which could be facilitated through shared architecture [21]. In Tanzania, there is demand to expand the system to other zoonoses and to further harmonize it within existing health and veterinary systems. Using phones as the building blocks for establishing and maintaining relationships with users can make systems participatory, empowering otherwise isolated frontline workers and, critically, can lead to the improved control and management of disease [22].

This work was approved by the Institutional Review Board of Ifakara Health Institute and the Medical Research Coordinating Committee of the National Institute for Medical Research of Tanzania (NIMR/HQ/R.8a/Vol.IX/946).

## Supporting Information

S1 Data Example data reported through the surveillance system presented in Fig 1. (XLSX)

S2 Data Data from usability study as shown in Fig 2A–2C and described in detail in S1 Text. (XLS)

S1 Table Helpline log enquiries categorized by form type. Data summarized in Fig 2D.
(DOCX)

S2 Table Compliance with PEP regimens during periods with and without SMS reminders. The period of monitoring prior to the SMS intervention was from 1 May 2011 to 18 Nov 2011, whilst the period of monitoring during the implementation of SMS reminders was from 19 Nov 2011 to 1 July 2012.
(DOCX)

S3 Table Mobile-phone–based form completion times and user attributes (DOCX)

S4 Table Mean records of animal bite injuries detected in each region captured by paper records from January 2005 to December 2010 and mobile-phone–based surveillance from January 2011 to January 2013. Numbers in brackets give the quarterly range reported for each region.
(DOCX)

S5 Table Breakdown of costs for setup and maintenance of mobile-phone–based and paper-based surveillance for rabies across 28 districts in southern Tanzania. One phone was allocated to each facility (clinic or livestock office), but four health workers per clinic and five livestock officers per office were trained. We assumed a phone replacement rate of 9% per annum. We only included costs for rabies- and/or animal-bite–specific forms that required collation for paper-based surveillance, and we considered that registers for recording patient data would be used for both surveillance types and did not include their costs. Similarly we did not consider depreciation of capital costs such as vehicles for distributing phones and registers, as we assumed the same assets would be required for both types of surveillance. Phone credit costs were minimal—sending a form cost <5 Tsh (<US$0.01) and surveillance personnel called back users contacting the helpline.
(DOCX)

S1 Text Additional methodological information. (DOCX)

## Abbreviations

GPRS: General Packet Radio Service
GSM: Global System for Mobile communication
IHI: Ifakara Health Institute
LMICs: low- and middle-income countries
mHealth: mobile-phone–based health
PEP: post-exposure prophylaxis
SMS: short message service or text messaging
