# Supplementary material for: Mobile Phones As Surveillance Tools: Implementing and Evaluating a Large-Scale Intersectoral Surveillance System for Rabies in Tanzania
Source: PLoS Med. 2016 Apr 12;13(4):e1002002. doi: 10.1371/journal.pmed.1002002 (PMC4829224; doi:10.1371/journal.pmed.1002002)
Supplement: S1 Table — Data summarized in Fig 2D. (DOCX) [file pmed.1002002.s003.docx]

**S1 Table**. Number of forms submitted by health workers (forms for bite patients including PEP administration) and livestock field officers (forms for reporting vaccination campaigns and suspect animals) and the number of helpline calls relating to each of these form types over a period of 18 months. Additional forms submitted by staff involved in system development and therefore familiar with the mobile phone application were excluded here. Annual vaccination campaigns initially only took place in the two major cities (Dar es Salaam and Morogoro) before the programme was rolled out to all districts, explaining the low number of submitted forms during 2011. These data are summarized in Figure 2D.

| **Dates** | **Bite / PEP** | | **Vaccination campaign** | | **Suspect animal** | |
| --- | --- | --- | --- | --- | --- | --- |
|  | **Helpline** | **Database** | **Helpline** | **Database** | **Helpline** | **Database** |
| 2011/04/05 – 2011/10/04 | 50 | 1945 | 27 | 97 | 18 | 122 |
| 2011/04/05 – 2012/04/04 | 54 | 2255 | 12 | 140 | 18 | 323 |
| 2012/04/05 – 2012/10/04 | 44 | 2444 | 22 | 1080 | 22 | 323 |
| **Total** | **148** | **6652** | **61** | **1317** | **58** | **768** |
