# Supplementary material for: Mobile Phones As Surveillance Tools: Implementing and Evaluating a Large-Scale Intersectoral Surveillance System for Rabies in Tanzania
Source: PLoS Med. 2016 Apr 12;13(4):e1002002. doi: 10.1371/journal.pmed.1002002 (PMC4829224; doi:10.1371/journal.pmed.1002002)
Supplement: S2 Table — The period of monitoring prior to the SMS intervention was from 1 May 2011 to 18 Nov 2011, whilst the period of monitoring during the implementation of SMS reminders was from 19 Nov 2011 to 1 July 2012. (DOCX) [file pmed.1002002.s004.docx]

**S2 Table. Compliance with PEP regimens during periods with and without SMS reminders.** The period of monitoring prior to the SMS intervention was from 1 May 2011 to 18 Nov 2011, whilst the period of monitoring during the implementation of SMS reminders was from 19 Nov 2011 to 1 July 2012.

|  | **Received dose** | | **Did not receive dose** | |
| --- | --- | --- | --- | --- |
| **PEP dose** | **pre-SMS** | **SMS period** | **pre-SMS** | **SMS period** |
| 2 | 1030 | 140 | 1119 | 85 |
| 3 | 881 | 113 | 1268 | 112 |
| 4 | 471 | 65 | 1678 | 160 |
| 5 | 160 | 29 | 1989 | 196 |
