# Supplementary material for: Mobile Phones As Surveillance Tools: Implementing and Evaluating a Large-Scale Intersectoral Surveillance System for Rabies in Tanzania
Source: PLoS Med. 2016 Apr 12;13(4):e1002002. doi: 10.1371/journal.pmed.1002002 (PMC4829224; doi:10.1371/journal.pmed.1002002)
Supplement: S3 Table — (DOCX) [file pmed.1002002.s005.docx]

**S3 Table. Final model of first-use form completion times in minutes analysed using linear regression.** Multiple R^2^ = 0.47. Asterisks represent levels of significance (* = p < 0.05, ** = p < 0.005, *** = p < 0.0005).

|  | **Transformations and categorical variables being compared to baseline** | **Estimate (in minutes) ± SE** | ***F*-statistic** | ***p*-value** |
| --- | --- | --- | --- | --- |
| Intercept |  | 18.8 ± 1.86 | -- |  |
| Sector | Livestock field officer | -1.35 ± 1.05 | 6.61 | 0.013* |
| Sex | Male | +3.55 ± 0.97 | 8.98 | 0.004** |
| SMS per day | *log_e_* transformed | -1.26 ± 0.52 | 19.68 | 3.9e-5*** |
| Time owned | *log_e_* transformed | -3.35 ± 1.07 | 8.48 | 0.005** |
| Use Internet | Yes | -3.17 ± 0.99 | 10.29 | 0.002** |
