# Supplementary material for: Mobile Phones As Surveillance Tools: Implementing and Evaluating a Large-Scale Intersectoral Surveillance System for Rabies in Tanzania
Source: PLoS Med. 2016 Apr 12;13(4):e1002002. doi: 10.1371/journal.pmed.1002002 (PMC4829224; doi:10.1371/journal.pmed.1002002)
Supplement: S4 Table — Numbers in brackets give the quarterly range reported for each region. (DOCX) [file pmed.1002002.s006.docx]

S4 Table. Mean records of animal bite injuries detected in each region captured by paper records from January 2005 to December 2010 and mobile phone-based surveillance from 2011 to January 2013. Numbers in brackets give the quarterly range reported for each region.

| **Regions** | **Paper records** | **Mobile phone records** |
| --- | --- | --- |
| Coast | 30 (0-107) | 61 (39-90) |
| Dar es Salaam | 22 (0-243) | 125 (81-185) |
| Lindi | 2 (0-21) | 54 (36-84) |
| Morogoro | 56 (0-187) | 191 (146-230) |
| Mtwara | 1 (0-1) | 79 (25-85) |
| Pemba (North & South) | 0 | 3 (0-6) |
