# Supplementary material for: Mobile Phones As Surveillance Tools: Implementing and Evaluating a Large-Scale Intersectoral Surveillance System for Rabies in Tanzania
Source: PLoS Med. 2016 Apr 12;13(4):e1002002. doi: 10.1371/journal.pmed.1002002 (PMC4829224; doi:10.1371/journal.pmed.1002002)
Supplement: S5 Table — One phone was allocated to each facility (clinic or livestock office), but four health workers per clinic and five livestock officers per office were trained. We assumed a phone replacement rate of 9% per annum. We only included costs for rabies- and/or animal-bite–specific forms that required collation for paper-based surveillance, and we considered that registers for recording patient data would be used for both surveillance types and did not include their costs. Similarly we did not consider depreciation of capital costs such as vehicles for distributing phones and registers, as we assumed the same assets would be required for both types of surveillance. Phone credit costs were minimal—sending a form cost <5 Tsh (<US$0.01) and surveillance personnel called back users contacting the helpline. (DOCX) [file pmed.1002002.s007.docx]

**S5 Table. Breakdown of costs for setup and maintenance of mobile phone-based and paper-based surveillance for rabies across Southern Tanzania.** One phone was allocated to each facility (clinic or livestock office), but four healthworkers per clinic and five livestock officers per office were trained. We assumed a phone replacement rate of 9% per annum.

We only included costs for rabies/animal bite specific forms that required collation for paper-based surveillance and average weekly delivery costs based on use of a regular bus service and taxi collection. We considered that registers for recording patient and vaccination data would be used for both surveillance types and did not include their costs. Similarly we did not consider depreciation of capital costs such as vehicles for distributing phones and registers, as we assumed the same assets would be required for both types of surveillance. Phone credit costs were minimal – sending a form cost <5 Tsh (< $0.01) and surveillance personnel called back users contacting the helpline.

|  |  | **Mobile phone surveillance** | | | **Paper surveillance** | |  |
| --- | --- | --- | --- | --- | --- | --- | --- |
| **Costs** | **Items** | Unit cost (TZS) | Quantity | Total (USD) | Unit cost (TZS) | Quantity | Total (USD) |
| **Capital costs** | Server | 6,250,000 | 2 | 8,065 | 6,250,000 | 1 | 4,032 |
|  | Computers | 1,147,500 | 3 | 2,221 | 1,147,500 | 2 | 1,481 |
|  | Phones | 150,000 | 192+18 | 20,323 |  |  | 0 |
|  | SIM cards | 1,500 | 192+18 | 203 |  |  | 0 |
| **Running costs** | Salaries: technicians & administrators | 14,400,000 | 3 | 27,871 | 9,600,000 | 2 | 12,387 |
|  | Phone credit | 6,000 | 192 | 743 |  |  | 0 |
|  | Paper forms |  |  |  | 100 | 12,480 | 805 |
|  | Printing fee |  |  |  | 50 | 12,480 | 403 |
|  | Distribution & collection |  |  | 0 | 15,000 | (96+24)x52 | 60,387 |
|  | Trouble shooting/ site visits | 1,500,000 | 6 | 5,806 | 1,500,000 | 6 | 5,806 |
| **Training** | Training team | 1,500,000 | 6x3 | 17,419 | 1,500,000 | 6x2 | 11,613 |
| once only | Training (per diem for users) | 50,000 | 192 | 6,194 | 50,000 | 192 | 6,194 |
| **Total** |  |  |  | 88,845 |  |  | 103,108 |
