# Supplementary material for: Mobile Phones As Surveillance Tools: Implementing and Evaluating a Large-Scale Intersectoral Surveillance System for Rabies in Tanzania
Source: PLoS Med. 2016 Apr 12;13(4):e1002002. doi: 10.1371/journal.pmed.1002002 (PMC4829224; doi:10.1371/journal.pmed.1002002)
Supplement: S1 Text — (DOCX) [file pmed.1002002.s008.docx]

**S1 Text. Additional Methodological Information**

### Additional details are provided on user training and on methods used to evaluate the usability of the mobile-phone—based surveillance system:

### User training

A training team comprising rabies personnel from the WHO Tanzania office, the Ministry of Health (MoHSW) and the Ministry of Livestock (MOLFD), alongside district officers with responsibility for health or veterinary workers and surveillance system instructors from Ifakara Health Institute (IHI), visited all districts to provide training to users. During training, users received an introduction to the system and instruction in the use of the mobile phones for their respective surveillance activities. User registration involved the collection of geographical and facility-based information so that these could be associated with data submitted by that user. Users were supplied with their own phone preloaded with credit and login details, a workbook manual and the helpline contact details.

**Evaluation of usability**

A usability study was conducted from June to December 2010 during training, just prior to full system deployment. A subset of trainees from each sector was recruited randomly, consisting of one livestock field officer (LFO) per district (N=27, female=2, male=25), and at least one health worker (HW) per district (N=40, female=24, male=16), giving a total sample size of N=67, with gender bias reflective of the gender distribution in these sectors. Participants were aged between 21 and 60. All users owned a mobile phone and all except 4 reported sending at least one SMS per day. Only 3 owned a computer and only 9 had an email address, while 43 reported never using either a computer or the Internet.

Participants were observed carrying out two tasks, with observations conducted during their very first encounter with the system: (1) logging in and downloading their relevant forms; and (2) completing and submitting the main form for their user category (a bite patient form or a dog vaccination campaign form respectively). The number of times that users were observed to get stuck and need prompting, and the total time required for each task (including both assistance and data entry time) were recorded. Participants also filled in a background questionnaire recording demographic information and self-reported data relating to their level of familiarity with mobile phones and other information technologies, as well as frequency of use (S2 Data).

### *Login and form download*

In a first stage, participants used the phone they had received, and logged in using their own details. All participants logged into the system successfully, with less than one quarter of LFOs and just over half the HWs requiring assistance. LFOs required slightly less time (mean=1.3 minutes) to log in and download their forms than HW (mean=1.8 minutes); the difference was statistically significant (Mann-Whitney U test, p < 0.05), and can probably be explained by factors such as network connectivity in more remotely stationed health facilities.

### *Form completion*

Once participants had logged onto the system, they used it to enter hypothetical data according to their sector of activity and the total time taken to complete this exercise was recorded. The total time taken serves as a measure of first-time system usability that captures the role of both the speed with which participants entered data and need for assistance. Participants required help between 0 and 7 times while completing the form (median 3 times for HWs and 2 for LFOs). HWs completed their form in 4-22 minutes (mean 11.9) while LFOs took 5-20 minutes (mean 9.8), with just under half of HWs and two-thirds of LFOs completing forms within 10 minutes (S2 Data). Although the upper limits on completion times appear long, these were measured during the first encounter with the system and included assistance time, so time spent would be expected to reduce with experience. For the 10 HWs and 7 LFOs who required no help, mean completion times were 8.5 and 7.7 minutes respectively. Around 25% and 50% of the variation in form completion time for HWs and LFOs respectively was explained by the number of occasions they required help.

We were interested in understanding why some users found it easier than others to complete the data entry exercise. Completing forms using the mobile phone system requires entering text using a mobile phone and general familiarity with digitised forms (e.g. drop-down menus, use of a submit button) and we hypothesised that familiarity with this kind of system would facilitate first-time usability. Data collection focused on these aspects of digital literacy as well as demographic variables. Specifically, we collected data on the following explanatory variables: (1) *demographic variables* (sector, age, gender, disabilities, educational level and recency of study); (2) *mobile phone variables* (SMS usage per day, time phone owned, monthly phone credit) and (3) *computer variables* (use of an email address, computer ownership, computer certification, and frequency of computer and Internet use). We began our analysis with a stage of univariable analysis, followed by multiple regression analysis.

#### Univariable analysis

In univariable analysis, age and SMS usage showed the clearest relationship with form completion times. Participants aged 40 or older took longer to complete forms than those under 40 (*t*-test on completion times, standardised to take account of sectorial differences, t = -3.16, df = 45.65, p < 0.01, Figure 2B, main text). We also observed a negative relationship between the number of SMS sent per day and relative completion times (Figure 2C, main text), and a scatter plot showed this relationship to be logarithmic (showing a decreasing advantage of sending additional SMS per day). We had expected level of education to be related to form completion times; however, level of education was not independent of sector (all LFOs had the same level of education) and sex (most LFOs were male and most HWs female) and patterns from univariable analysis were unclear. Six participants recorded sight problems (requiring glasses); however, median completion times did not differ significantly between these individuals and those without sight problems and we did not consider this further (Mann-Whitney U test, W = 211.5, p = 0.54).

#### Multivariable analysis

In this stage of data analysis we constructed a linear regression model with completion time as the response variable, controlling for sector throughout the analysis (veterinary forms were shorter). From each of the demographic, mobile phone and computer categories of explanatory variables, we initially included the variable with the highest correlation with completion time in the univariable analysis (age, logged SMS per day, Internet use). Proceeding one category at a time, we then added variables sequentially, retaining those with coefficients significantly different from zero and that resulted in improvements in explanatory power (adjusted R^2^). We also tested variables that met these criteria as replacements for the initial variables, and the addition of interaction terms between variables with correlation coefficients of >0.25 in the univariable analysis. Once a proposed final model was obtained, robustness testing was conducted by removing one variable at a time (to check for variable inclusion separately from variable categories). The final model was tested for outliers, normality of residuals, heteroscedasticity, as well as functional form and omitted variable bias, and we found the model to be consistent with assumptions.

The final model explained 47% of the variation in completion times (details provided in S3 Table). Overall, the regression analysis showed that a large proportion of the variation in first-time system usability during data entry could be explained by the answers to fairly simple questions relating to digital literacy. Individuals who regularly sent SMS messages, who had owned a mobile phone for longer, and who had used the Internet, completed the data entry task more rapidly. After accounting for phone and computer variables, age was not included in the final regression model, suggesting that increasing familiarity should at least partially compensate for age-related effects. Nonetheless, the relationship between age and first-time usability in the univariable analysis suggests that it may serve as a proxy for digital literacy. Simple assessment of these factors can help trainers to anticipate assistance requirements during training and allocate sufficient time when introducing mobile phones as health tools.
